# Growth Factor Engineering Strategies for Regenerative Medicine Applications

**DOI:** 10.3389/fbioe.2019.00469

**Published:** 2020-01-21

**Authors:** Xiaochen Ren, Moyuan Zhao, Blake Lash, Mikaël M. Martino, Ziad Julier

**Affiliations:** ^1^European Molecular Biology Laboratory Australia, Australian Regenerative Medicine Institute, Monash University, Melbourne, VIC, Australia; ^2^Department of Biological Engineering, Massachusetts Institute of Technology, Cambridge, MA, United States

**Keywords:** growth factors, protein engineering, regenerative medicine, biomaterials, extracellular matrix

## Abstract

Growth factors are critical molecules for tissue repair and regeneration. Therefore, recombinant growth factors have raised a lot of hope for regenerative medicine applications. While using growth factors to promote tissue healing has widely shown promising results in pre-clinical settings, their success in the clinic is not a forgone conclusion. Indeed, translation of growth factors is often limited by their short half-life, rapid diffusion from the delivery site, and low cost-effectiveness. Trying to circumvent those limitations by the use of supraphysiological doses has led to serious side-effects in many cases and therefore innovative technologies are required to improve growth factor-based regenerative strategies. In this review, we present protein engineering approaches seeking to improve growth factor delivery and efficacy while reducing doses and side effects. We focus on engineering strategies seeking to improve affinity of growth factors for biomaterials or the endogenous extracellular matrix. Then, we discuss some examples of increasing growth factor stability and bioactivity, and propose new lines of research that the field of growth factor engineering for regenerative medicine may adopt in the future.

## Introduction

Growth factors (GFs) are molecules capable of stimulating a variety of cellular processes including cell proliferation, migration, differentiation and multicellular morphogenesis during development and tissue healing. Therefore, they have raised a lot of hope for regenerative medicine applications and several products based on growth factors have been developed (Table 1A). Nevertheless, GF-based therapies present limitations. For example, high levels of proteolytic activity *in vivo* leads to poor GF stability and short half-life (Mitchell et al., 2016). Thus, multiple administrations and/or supraphysiological doses are often necessary to sustain an effective concentration of GFs at the delivery site, resulting in high cost and adverse effects (Table 1B). Side effects and poor effectiveness are mainly linked to sub-optimal delivery systems and lack of control over GF signaling. These issues in clinically available products emphasize the need to design new strategies allowing the use of lower and localized doses of GFs where delivery and signaling are tightly controlled.

**Table 1A:** Recombinant GF-based products for regenerative medicine applications.

**Product**	**GF**	**Delivery system**	**Target tissue/disease**	**Approved authority**	**References**
Augment® Bone Graft	PDGF-BB	Beta-tricalcium phosphate	Ankle fusion, hindfoot	FDA	FDA, 2015a
Increlex®	IGF-1	Subcutaneous Injection	Primary IGF-1 deficiency	FDA	FDA, 2005; National Drug Strategy, 2006
Infuse® Bone Graft	BMP-2	Collagen sponge	Spinal fusion, bone regeneration	FDA	James et al., 2016
Kepivance®	FGF-7 (KGF)	i.v. injection	Gastrointestinal injury	FDA	FDA, 2015b
OP-1® Putty	BMP-7	Bovine bone-derived collagen	Spinal fusion, bone regeneration	FDA	Okabe et al., 2013
PELNAC®	FGF-2 (bFGF)	Collagen sponge	Bedsores, cutaneous ulcers	Pharmaceuticals and Medical Devices Agency (Japan)	Kakudo et al., 2019
REGEN-D®	EGF	Cellulose gel	Foot ulcer	Ministry of Food and Drug Safety (South Korea)	Frew et al., 2007
Regranex®	PDGF-BB	Sodium carboxymethylcellulose-based topical gel	Chronic diabetic wound	FDA	FDA, 2008
Citrix® CRS	TGF-β1	Topical	Aged skin		Aldag et al., 2016

*BMP, bone morphogenetic protein; EGF, epidermal growth factor; FGF, fibroblast growth factor; HGF, hepatocyte growth factor; IGF, insulin-like growth factor; PDGF, platelet-derived growth factor; TGF, transforming growth factor; FDA, U.S. Food Drug Administration*.

**Table 1B:** Common GFs in regenerative medicine.

**GFs**	**Desired function(s)**	**Half-life in blood**	**Side-effect(s) in humans**	**References**
BMP-2	Osteogenic factor	1–4 h	Ectopic bone formation, abnormal osteogenesis, inflammatory complications, urogenital events, wound complications, increase cancer risk	Carragee et al., 2013; Carreira et al., 2014; James et al., 2016
BMP-7	Osteogenic factor, regulate proliferation of neural progenitor cells	1–4 h	Not reported	Calori et al., 2009; Carreira et al., 2014; Kowtharapu et al., 2018
EGF	Stimulates proliferation and differentiation of epithelial cells	<1 min	Not reported	Mitchell et al., 2016
FGF-2	Stimulates proliferation and differentiation of various cell types, angiogenesis	7.6 h	Not reported	Beenken and Mohammadi, 2009; Maddaluno et al., 2017
HGF	Stimulates epithelial cell proliferation and morphogenesis, angiogenesis	3–5 min	Not reported	Yu et al., 2007; Nakamura et al., 2019
IGF-1	Enhances neuronal growth, myelination, endometrial epithelial cell proliferation, inhibition of cell apoptosis	3–5 h	Not reported	Leroith et al., 1992; Wang et al., 2018
PDGF-BB	Proliferation of various cell types, extracellular matrix synthesis, vascularization	30 min	Increase cancer risk	Jin et al., 2008; Saika et al., 2011; Mao and Mooney, 2015; Yamakawa and Hayashida, 2019
FGF-7 (KGF)	Epithelium morphogenesis, re-epithelialization	4–6 h	Enhance epithelial tumor cell growth	FDA, 2004a,b; Yamakawa and Hayashida, 2019
TGF-β1	Differentiation of bone-forming cells, antiproliferative factor for epithelial cells	>100 min (latent form) 2–3 min (active form)	Not reported	Hermonat et al., 2007; Lee et al., 2011; Tian et al., 2011
VEGF-A	Angiogenic factor	30 min	Edema, systemic hypotension	Simons and Ware, 2003; Stefanini et al., 2008; Yamakawa and Hayashida, 2019

*BMP, bone morphogenetic protein; EGF, epidermal growth factor; FGF, fibroblast growth factor; HGF, hepatocyte growth factor; IGF, insulin-like growth factor; PDGF, platelet-derived growth factor; TGF, transforming growth factor; VEGF, vascular endothelial growth factor*.

Numerous strategies have been explored, in particular with the design of biomaterial-based delivery systems, focusing on engineering biomaterials instead of modifying GFs (Wang et al., 2017). In addition, interesting approaches have emerged to enhance the stability and bioactivity of GFs (Niu et al., 2018). In this review, we will focus on strategies aiming at engineering the GF itself. We first describe approaches to control GF half-life as well as spatial and temporal release. Then, we discuss various strategies to modulate GF signaling at the receptor level.

## Engineering GFs to Control Spatial and Temporal Presentation

**Figure 1:**
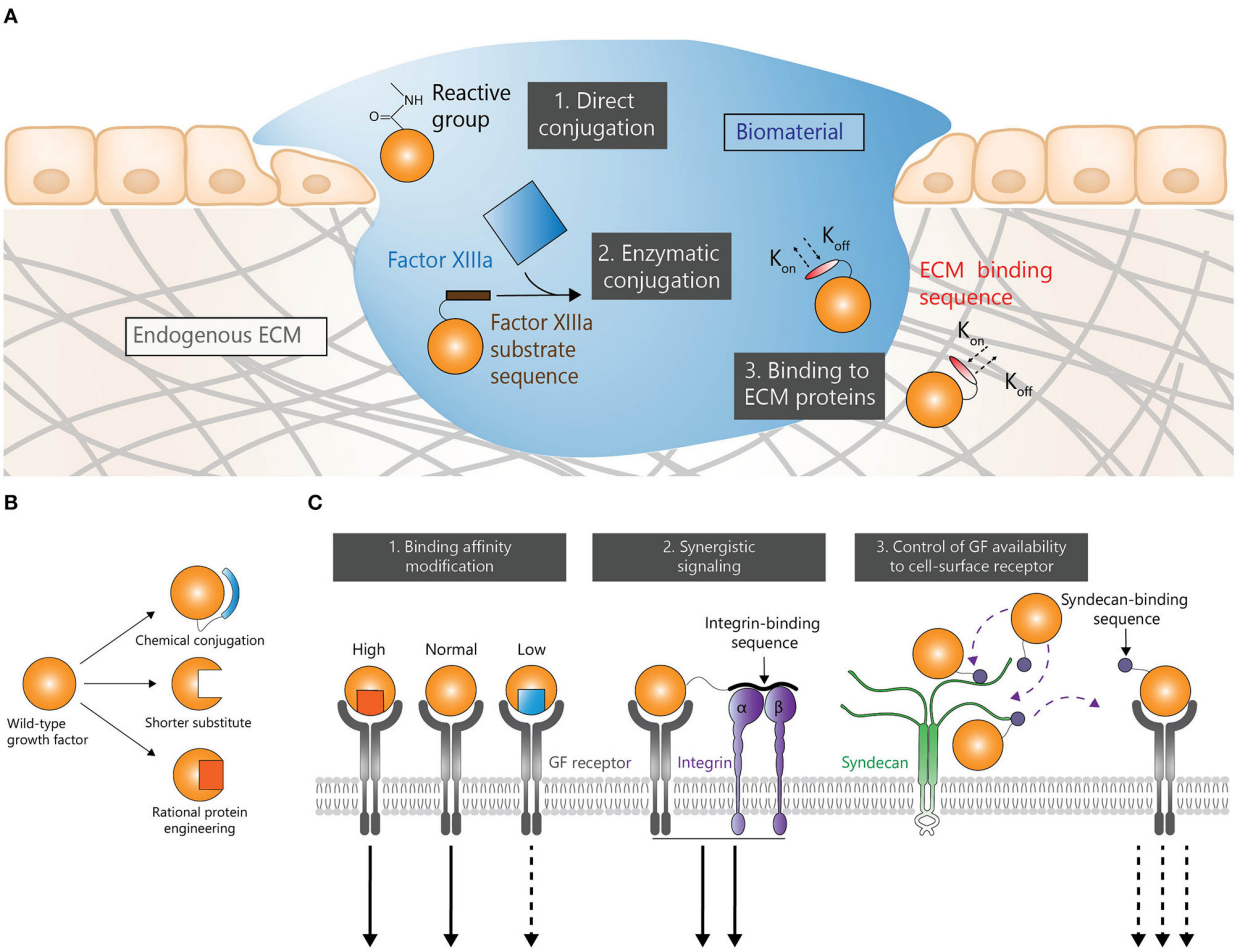
GF engineering strategies in regenerative medicine. Strategies to control spatial and temporal presentations **(A)**, stability **(B)**, and signaling **(C)**.

Biomaterial-based delivery is a common strategy to efficiently deliver GFs. Immobilizing GFs within a biomaterial (Figure 1A) gives the possibility to achieve a sustained release and a localized delivery. Such approaches may considerably reduce the need for multiple doses and potentially reduce adverse effects. Therefore, various methods have been explored to enhance interactions between GFs and biomaterials.

### Engineering GFs to Be Covalently Bound to Biomaterials

Covalent conjugation is a common strategy to immobilize GFs in biomaterials. In that setting, GF release depends on biomaterial degradation and/or cleavage (hydrolytic and enzymatic) of the bond between GFs and biomaterials. Additionally, this strategy can also address low stability problems as it may reduce the exposure of GFs to a proteolytic microenvironment at the delivery site. Strategies to bind GFs to biomaterials via reactive chemical groups have been widely used (Cabanas-Danés et al., 2014). For instance, crosslinking via 1-ethyl-3-(3-dimethyl aminopropyl) carbodiimide (EDC) and N-hydroxy-succinimide (NHS) has been extensively used, due to its simplicity, low cost, mild reaction conditions, and biocompatibility (approved by the USFDA) (Grabarek and Gergely, 1990; Masters, 2011). For example, EDC-mediated immobilization of bone morphogenetic protein-2 (BMP-2) onto a polyelectrolyte multilayer film successfully promoted bone regeneration in critical-size rat femoral defect (Bouyer et al., 2016). Although EDC/NHS has extensive merits, this chemistry links primary amines with carboxylic acids in an inherently random fashion, and not necessarily always at the terminal reactive groups. The inability of this approach to recognize the difference between terminal reactive groups and reactive groups within the protein backbone may hinder the recognition of GFs by their receptors and extracellular matrix (ECM) components (Mitchell et al., 2016).

Enzymatic conjugation is another interesting method to bind GFs to biomaterials. For example, GFs have been engineered with a transglutaminase substrate sequence derived from α_2_-plasmin inhibitor (α_2_PI_1−8_) (Schense and Hubbell, 1999). This strategy allows the engineered GFs to react with lysine residues via the transglutaminase factor XIIIa. GFs such as BMP-2, vascular endothelial growth factor A (VEGF-A), platelet derived growth factor AB (PDGF-AB) and insulin-like growth factor 1 (IGF-1) have been cross-linked to fibrin using this approach (Schmoekel et al., 2005; Sacchi et al., 2014; Mittermayr et al., 2016; Vardar et al., 2018). For instance, BMP-2 with α_2_PI_1−8_ fused at its N-terminus was delivered in fibrin matrices in critical-size craniotomy in rats (Schmoekel et al., 2005). Here, the engineered BMP-2 induced significantly higher bone formation compared to wild type BMP-2 after 3 weeks. Similarly, a fusion protein consisting of α_2_PI_1−8_ and VEGF-A delivered in fibrin induced a functional angiogenesis and promoted regeneration in ischemic hind limb wound models in rodents (Sacchi et al., 2014). Aberrant vessel formation and vascular hyperpermeability are adverse effects associated with the uncontrolled delivery of VEGF-A which induces a burst signaling. However, it was demonstrated that low doses (0.01– 5 μg/mL) of α_2_PI_1−8_-VEGF-A promotes normal angiogenesis. Following the same approach an α_2_PI_1−8_-VEGF-C fusion was engineered to stimulate local lymphangiogenesis upon delivery in a fibrin matrix (Güç et al., 2017). The lymphangiogenesis induced by the fibrin-binding VEGF-C promoted wound healing in diabetic model as shown by extracellular matrix deposition and granulation tissue thickening. The same strategy can be used to cross-link GFs to polyethylene glycol (PEG) hydrogel as multiarm PEG molecules can be functionalized with factor XIIIa substrates to drive its crosslinking and incorporation of α_2_PI_1−8_-bearing proteins (Ehrbar et al., 2007).

Another approach to combine GFs and biomaterials is to directly create fusion proteins consisting of GFs with ECM proteins. For example, a biopolymer based on the ECM protein elastin was fused to VEGF-A (ELP-VEGF) with the objective to treat preeclampsia, a hypertensive syndrome that originates from an improperly vascularized and ischemic placenta (Logue et al., 2017). Here, ELP-VEGF reduced hypertension in a placental ischemia rat model and did not cross the placental barrier, reducing the risk of adverse effects on fetal development. Although covalent binding of GFs to biomaterial has proved to be an effective strategy it is nevertheless dependent on the biocompatibility of the used biomaterials. In addition, as the system requires both a biomaterial and an engineering protein it may complicate the pathway to approval or increase the cost.

### Engineering GFs for Non-covalent Interaction to Biomaterials and Endogenous ECM

GFs can be immobilized to the ECM or ECM-derived biomaterials through affinity binding by the introduction of an ECM-binding sequence or domain at either terminus of the GF. The strategy presents the advantage of giving modified GFs the ability to bind the endogenous ECM where the GF is delivered, in some cases allowing to forgo the use of exogenous biomaterials altogether. Such approach allows GFs to be more readily available for resident cells by being immobilized in the local ECM instead of having to be released by biomaterials. In addition, the simplicity of biomaterial-free delivery systems could lead to a higher cost-effectiveness. However, the effectiveness of these strategies may depend on the local ECM composition.

As one of the most abundant ECM proteins, collagens represent good binding targets for engineering GFs for delivery to collagen-rich tissues. For example, a bacterial collagen-binding domain (CBD), was fused to fibroblast growth factor-2 (FGF-2), allowing improved bone formation in a spinal fusion model (Inoue et al., 2017). In another study, CBD-fused FGF-2 showed the ability to induce significantly higher mesenchymal cell proliferation and callus formation in a mice fracture model compared to wild-type FGF-2 (Sekiguchi et al., 2018). Similarly, a CBD-fused hepatocyte growth factor (HGF) delivered via hydrogel improved recovery after spinal cord injury in mice compared to wild-type HGF (Yamane et al., 2018).

In order to engineer GFs with stronger binding to collagen, a library of random sequences was conjugated to VEGF-A and selected *in vitro* for their binding affinity to collagen (Park et al., 2018). This method presents the advantage of identifying CBDs tailor-made for a specific GF as large GFs may affect the binding of generic CBDs to collagen. The resulting engineered collagen-binding VEGF-A stimulated angiogenesis in skin wounds and infarcted myocardiums in mice.

Natural interactions between the ECM and GFs are crucial for tissue healing (Schultz and Wysocki, 2009) as many GFs have the ability to bind ECM proteins to some extent (Macri et al., 2007; Sawicka et al., 2015). These interactions often occur between the heparin-binding domains of ECM proteins and heparin-binding GFs (Martino et al., 2013). For example, PIGF-2_123−144_, a placental growth factor-2 (PlGF-2)-derived ECM-binding domain, promiscuously binds multiple ECM proteins with high affinity (Martino et al., 2014). The sequence was fused to VEGF-A, PDGF-BB, and BMP-2, and the engineered variants showed the ability to bind several ECM proteins with much higher affinity (i.e. super-affinity) compared to their wild-type counterparts. Super-affinity GFs contributed to improved therapeutic efficacy in murine models of chronic wounds and bone regeneration (Martino et al., 2014). Moreover, this approach significantly reduced the vascular hyperpermeability induced by VEGF-A.

In hard bone tissues, the ECM exists in the form of either a collagen-rich organic phase, or a calcium-phosphate (Ca-P) mineral phase (mainly hydroxyapatite) (Boonrungsiman et al., 2012). However, most GFs do not express mineral-binding domains, limiting natural interactions between the bone ECM and GFs. To overcome this limitation, several studies have explored the introduction of mineral-binding domains into GFs. Indeed, some bone ECM proteins such as osteocalcin (OC) can bind to hydroxyapatite (HA) minerals, the major inorganic component of bone tissue, through a C-terminal sequence (Dowd et al., 2003). For instance, a FGF-2-OC fusion protein displayed a significantly stronger HA-binding affinity than wild-type FGF-2 and retained its bone repair and regeneration properties (Jeon and Jang, 2009).

## Stability Enhancement

As mentioned earlier, some of the major limitations of GFs are their poor stability in physiological environment and rapid enzymatic degradation. The following section focuses on modifying the thermal stability and protease-resistance of GFs (Figure 1B), although other factors not detailed here can also reduce the stability of GFs. It is however noteworthy that decreasing the natural clearance rate of GFs from the body may requires additional side-effects monitoring.

### Improving Thermal Stability

A common method to improve thermal stability of GFs is attaching a stable polypeptide or molecule, such as PEG, onto either terminal of the protein. The addition of PEG to GFs, or PEGylation, has been successfully applied clinically as the method of choice for extending protein half-life due to its flexibility, hydrophilicity, and low toxicity. To date, the USFDA has approved more than 15 PEGylated protein therapeutic products, and more are under development (Ramos-de-la-Peña and Aguilar, 2019). For instance, IGF-I is a mitogenic GF capable of stimulating anabolic processes in tissue repair and regeneration but is limited by its short half-life. Thus, a modified IGF-I was engineered through site-specific PEGylation and remained stable up to 8 h when exposed to 10% human serum. Moreover, this engineered IGF-1 showed a 3-fold increase in serum stability after 18 h incubation compared to wild-type IGF-I (Braun et al., 2018). Additionally, although PEGylated molecules often show a reduced bioactivity (Simone Fishburn, 2008; Braun et al., 2018), the site-specific nature of the modification allowed the preservation of IGF-I activity. Other molecules can also enhance the half-life of GFs. For example, conjugation of apolipoprotein A-I to the C-terminus of FGF-19 led to a 10-fold increase in circulating half-life (Alvarez-Sola et al., 2017).

Genetic modification is another effective approach to reinforce thermal stability of GFs. Indeed, the amino acid sequence of GFs can be edited to reinforce the local conformation and strengthen their tertiary structure. For instance, by introducing a triple mutation to FGF-1 increasing van der Waals forces and steric strains, a 21.5°C increase in denaturation temperature compared to wild-type FGF-1 was observed (Zakrzewska et al., 2005; Szlachcic et al., 2009). In addition, disulphide bonds are critical components of the protein structure which can greatly enhance its stability, thereby promoting bioactivity (Wedemeyer et al., 2000). In that regard, FGF-1 contains an unpaired cysteine at position 83 contributing to its poor stability. Therefore, by applying site-specific mutagenesis, Ala66 was replaced by a cysteine to introduce a disulphide bond between position 66 and 83. This variant showed a 14-fold increase in half-life and 10-fold increase in mitogenic activity (Kobielak et al., 2014).

### Reducing Extracellular Proteolytic Degradation

As an indispensable element during wound healing and regeneration, proteases regulate the clearance of damaged proteins and matrix and facilitate cell infiltration (Schultz and Wysocki, 2009). However, in some cases, proteases impair tissue repair through excessive tissue degradation. Especially in chronic wounds, stimuli such as bacteria, foreign material, and impaired tissue lead to elevated and prolonged presence of proteases at the wound site. This aberrant expression of tissue-degrading enzymes results not only in poor healing outcomes, but in the degradation of pro-regenerative growth factors (Schultz and Wysocki, 2009; Harding et al., 2011; McCarty and Percival, 2013). Therefore, altering the protease-sensitive sites that naturally occurs within GFs can be an efficient method to enhance their activity. For instance, two mutations introduced at a known cleavage site in FGF-1, has demonstrated to significantly increase the proteolytic resistance of the protein up to 100-fold (Schultz and Wysocki, 2009). A similar strategy has been used for VEGF-A (Lauer et al., 2002; Traub et al., 2013).

## Modifying GFs Signaling and Functionality

The signaling properties of GFs can be modified to enhance their regenerative activity. The next section focuses on different approaches that attempt to modify the sequence or the structure of GFs to promote their function (Figure 1C), thereby effecting similar or altogether different responses at lower doses. Although those strategies have the potential to produce highly effective modified GFs, they may require longer development as the effects of modified signaling may be less predictable than those of improved delivery or stability.

### Binding Affinity Modification

Binding affinities between GFs and their receptors can be modified to induce alternative signaling (Spangler et al., 2015). Whether higher or lower binding affinity is required is highly dependent on the receptor-ligand system and can lead to enhancement or abrogation of signaling in either case. For instance, site-directed mutagenesis to the residues Ile 38, Glu 51 and Leu 52 of epidermal growth factor (EGF) produced mutants with up to 30 fold higher affinity for EGF receptor (EGFR) (Cochran et al., 2006; Lahti et al., 2011). However, high affinity ligands may trigger a fast receptor internalization and degradation abrogating their signaling. Inversely, low affinity ligands may preserve the receptor leading to a longer lasting signaling (Zaiss et al., 2015).

In the EGF-EGFR signaling pathway, ligands which dissociate from the receptor within the endosome preferentially sort toward recycling rather than lysosomal fusion. Ligands that remain bound are degraded with the receptor, leading not only to receptor downregulation, but ligand depletion. Therefore, the sustained signaling response from EGF mutant with lower binding affinity, may elicit greater cell proliferation (Reddy et al., 1996; Zaiss et al., 2015).

### Synergistic Signaling

Although protein engineering allows numerous ways to engineer delivery mechanisms and systemic or local degradation kinetics, perhaps the most unique aspect of this field is the ability to confer non-canonical functionality to GFs for the purposes of promoting regeneration. In terms of circumventing the clinical limitations of GFs, there are several stages in the process that can be modified. GFs can be engineered to engage alternative signaling pathways through the creation of hybrid proteins. For example, there is significant crosstalk between integrin signaling and growth factor receptors such as VEGFR-2 and EGFR (Mahabeleshwar et al., 2008; Brizzi et al., 2012). The integrin-binding type III 10^th^ repeat of fibronectin (FNIII10) was fused to VEGF-A to create a bi-functional engineered protein (FNIII10-VEGF-A) with the ability to bind both VEGFR-2 and integrin αvβ3 (Traub et al., 2013). Surfaces coated with FNIII10-VEGF induced a significantly higher cell attachment and spreading of endothelial cells compared to FNIII10 or VEGF-A_165_. However, even though FNIII10-VEGF immobilized in a fibrin matrix enhanced angiogenesis in a diabetic mouse skin wound model compared to soluble VEGF-A, the angiogenic response was reduced compared to the one induced by fibrin-immobilized VEGF-A. This suggests that although the crosstalk that exist between integrins and GF receptors could be used to induce improved regeneration, balancing the contribution of each signal is critical to optimize the desired effect.

### Control of GFs Availability to Cell-Surface Receptor

Although the affinity of a GF for its receptor is critical in defining its effects, GF signaling can be controlled upstream of the GF-receptor interaction. Indeed, the availability of a GF for its receptor can be modulated not only by the ECM (Briquez et al., 2016) but also on the cell surface through binding to heparin sulfate proteoglycans (Rogers and Schier, 2011) such as syndecans (Kwon et al., 2012). In order to use the ability of syndecans to modulate GFs signaling, a syndecan-binding domain (SB) from laminin subunit α1 was fused to PDGF-BB (PDGF-BB-SB) and VEGF-A (VEGF-A-SB) to create syndecan-binding variants (Mochizuki et al., 2019). The controlled availability of PDGF-BB-SB and VEGF-A-SB for their cognate receptor on mesenchymal stem cells and endothelial cells, respectively, led to a long-lasting tonic signal as opposed to the short-lived burst signal induced by their wild type counterparts. Moreover, PDGF-BB-SB induced a significantly improved bone regeneration in a mouse bone defect model compared to PDGF-BB while VEGF-A-SB successfully improved skin wound healing in diabetic mice compared to wild-type VEGF-A. Interestingly, the engineered GFs abrogated common side-effects associated with clinical use of PDGF-BB and VEGF-A, respectively cancer risks and vascular permeability.

## Conclusion and Perspectives

Due to their critical role in tissue development and healing, GFs are ideal candidates for developing regenerative medicine therapies, but examples of successful clinical applications of GFs are still scarce. Throughout evolution, GFs have been selected to carry out specific tasks in specific environments, while being produced as needed by cells. However, their use in regenerative medicine requires to push the boundaries of their natural functions and is therefore met with limitations, such as instability or rapid diffusion from the delivery site. Trying to circumvent those limitations by delivering multiple supraphysiological doses has proven unsafe and thus highlights the need for the development of novel delivery systems (Niu et al., 2018).

The GF engineering approach is promising and generally aims at modulating the bioactivity and stability of GFs or controlling their interaction with biomaterials and the endogenous ECM. These different approaches present the advantage of being compatible with one another. It would be indeed possible to increase the bioactivity and stability of a GF while simultaneously increasing its affinity for the ECM or a biomaterial, opening the door to numerous new technologies. It is however important to note that as promising as these new technologies are, none of them are likely to represent a universal solution. Most GFs have their particular set of limitations and will require the development of new approaches for regenerative medicine to fully tap in their potential.

Future strategies in GF-based regenerative therapies may benefit by embracing a more comprehensive approach to tissue repair, as it is now evident that the immune system plays a critical role in the regenerative process (Julier et al., 2017; Larouche et al., 2018). Thus, future strategies may benefit from the co-delivery of GFs and immunomodulators or the development of multifunctional fusion proteins, with the ability of promoting morphogenesis while modulating the immune system. Moreover, most of the delivery strategies that we covered here aimed at improving the GF release and stability at the delivery site. However, several conditions, in particular ischemic injuries such as stroke or myocardial infarcts, occur at sites that are difficult to reach without invasive surgical procedures. Therefore, one of the main challenges that lies ahead is the development of engineered GFs with the ability to target distant sites.

## Author Contributions

XR, MZ, BL, MM, and ZJ wrote the manuscript. XR, MM, and ZJ made the tables and figure. MM and ZJ supervised the writing.

### Conflict of Interest

The authors declare that the research was conducted in the absence of any commercial or financial relationships that could be construed as a potential conflict of interest.
